# Effect of Food Matrix on Regulation of Intestinal Barrier and Microbiota Homeostasis by Polysaccharides Sulfated Carrageenan

**DOI:** 10.3390/foods14040635

**Published:** 2025-02-14

**Authors:** Xuke Shang, Juanjuan Guo, Peilin Chen

**Affiliations:** 1 College of Oceanology and Food Sciences, Quanzhou Normal University, Quanzhou 362000, China; sxk7952024@163.com (X.S.); cplfst@163.com (P.C.); 2 College of Food Science, Fujian Agriculture and Forestry University, Fuzhou 350002, China; 3 Fujian Province Key Laboratory for the Development of Bioactive Material from Marine Algae, Quanzhou 362000, China; 4 Key Laboratory of Inshore Resources Biotechnology, Quanzhou Normal University, Quanzhou 362000, China

**Keywords:** carrageenan, casein, sulfate group, intestinal barrier, gut microbiota, *Oscillibacter*

## Abstract

Carrageenan (CGN) has side effects on the intestinal barrier. Damage to the intestinal barrier is associated with exposure to sulfate groups. Food matrix has significant influence on the exposure quantity of sulfate groups and conformation in κ-CGN, but the corresponding side effects are not reported specifically. This study aimed to explore the regulatory effect of κ-CGN dissolved in aqueous (κ-CGN) and in 3% casein (κ-carrageenan-casein, κ-CC) on the intestinal barrier and microbiota homeostasis. Research has shown that both κ-CGN and κ-CC can induce different extents of intestinal barrier damage through disrupting microbiota homeostasis. Importantly, κ-CGN in casein with lower sulfate groups content was found to repair the intestinal barrier injury induced by an equivalent dose of κ-CGN aqueous through increasing the abundance of *Oscillibacter* and decreasing *Weissella*. These alleviating effects were reflected in lower levels of tumor necrosis factor (TNF)-α and C-reaction protein (CRP), higher levels of interleukin (IL)-10, raised secretion of mucus and goblet cells, and improved expression of epithelial cell compact proteins zonula occluden (ZO)-1 and mucin protein 2 (MUC2). This study states that κ-CGN in casein has a positive regulatory effect on the intestinal barrier damage compared to in aqueous solution, which can provide guidance for processing and utilization of CGN.

## 1. Introduction

Carrageenan (CGN) is a high-molecular-weight sulfated polysaccharide extracted from the edible marine algae Rhodophyta [1]. The basic unit of CGN is repeated galactose and 3,6-dehydrated galactose alternately connected via α-1,3 and β-1,4 glycosidic linkages [2,3]. According to the amount and position of sulfate groups, CGN is generally classified into three major types, including kappa (κ-), iota (ɩ-), and lambda (λ-). Kappa-carrageenan (κ-CCN) is widely used as food additive to improve food texture due to its properties, such as thickening, gelling, as well as stable and marked chemical reactivity [4]. However, the side effects of κ-CGN on intestinal barrier disruption have been controversial since the 1970s [5]. Considerable evidence shows that exposure to 0.1–5% CGN solution can cause varying degrees of ulceration or damage in the intestines of animals, such as mice, rabbits, and monkeys [6,7,8,9,10]. Conversely, Weiner in 2015 suggested 2250 ppm CGN dissolved in infant formula did not cause significant effects on body weight or serum inflammatory factors in pre-weaning piglets [11]. At the same time, the Joint Expert Committee on Food Additives (JECFA) reported that CGN did not cause damage to the intestines when contained in infant milk, and emphasized that food matrix has an important impact on the consumption of food additives [12]. Regarding the side effects of CGN, the focus of controversy is whether it causes damage to the intestinal mucus barrier, such as colonic edema, inflammatory cell infiltration, increased intestinal permeability, goblet cell loss, and lower secretion of intestinal acid mucus [13,14,15]. At present, most studies focused on the mechanism of CGN of the intestinal barrier disruption of drinking water as a matrix, but the effect of a vehicle on the intestinal barrier of CGN is worthy of further study [16,17,18].

Furthermore, recent investigations indicated that CGN aqueous solution causes damage to the homeostasis of the host’s intestinal microbiota and promotes the occurrence of intestine barrier damage [19]. Munyaka et al. [20] in 2016 suggested that 1% CGN in a piglet model changed the composition and structure of the gut microbiota, leading to bacterial dysbiosis. In the following year, a study showed that CGN can decrease the abundance of the anti-inflammatory bacterium, *Akkermansia muciniphila*, which is a mucin-degrading bacterium that is inversely associated with gut barrier damage [18]. In 2020, Mi et al. [21] revealed that the disruption of the epithelial barrier induced by CGN by a high-fat diet was related to changes in the composition of the gut microbiota, increasing the abundance of pathogenic bacteria, such as *Alistipes* and *Bacteroides*. Thus, the imbalance of intestinal microbiota is considered to have a solid relation with epithelial barrier damage.

**Figure 1:**
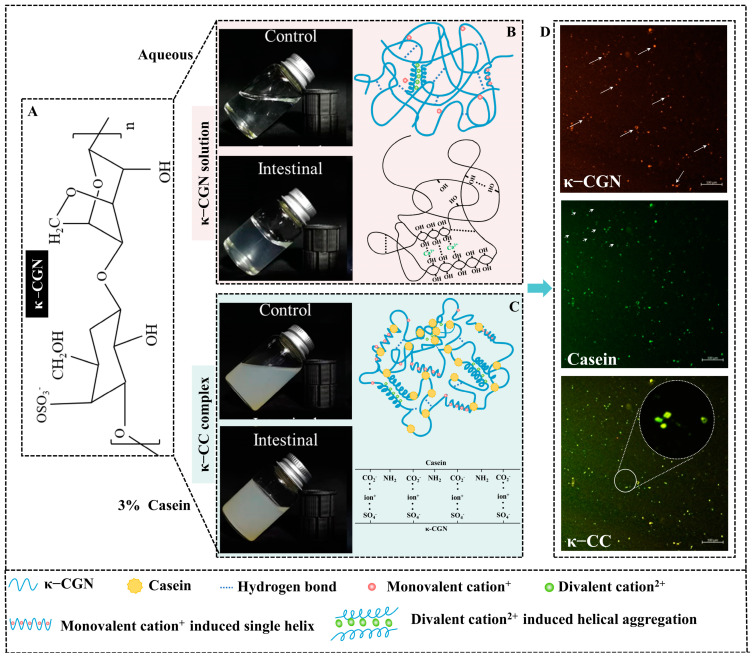
The conformational characterizations of the κ-CGN and κ-CC. (**A**) The basic unit structures of κ-CGN. (**B**) Physical diagram and conformational characterizations of the κ-CGN in the simulated intestinal phase. (**C**) Physical diagram and conformational characterizations of the κ-CC in the simulated intestinal phase. (**D**) Confocal laser scanning microscopy images. The arrows indicate the microscopic morphological features of the sample observed under laser confocal microscopy. κ-CGN appears green while casein is red/orange.

CCN as sulfated polysaccharide contains 15–40% of ester sulfate group [22]. The sulfate contained in food plays an important role in human metabolism [23]. SO_4_^2−^ was released during the degradation of sulfate, stimulating the increase in sulfate-reducing bacteria in the gut [24]. Then, sulfates were reduced by bacteria to potentially toxic and harmful metabolites, such as hydrogen sulfide (H_2_S) [25]. The increased levels of H_2_S in the gut have been associated with inflammatory bowel disease (IBD) formation. Excessive exposure to H_2_S can be detrimental to the host, increasing mucosal permeability and blocking butyrate metabolism [26]. Our previous experiments indicated that κ-CGN in aqueous solution existed as a random coil and exhibited a self-spiral disordered state in intestinal digestion. Casein and κ-CGN formed a complex (κ-carrageenan-casein, κ-CC) via electrostatic interactions and hydrogen bonds and maintained a helical structure (Figure 1). Most importantly, it can be observed that κ-CGN in casein reduced the exposure quantity of the free sulfate group of κ-CGN in aqueous solution significantly [27,28,29]. The free sulfate group’s content differences of κ-CGN in different vehicles may contribute to the different inflammatory attributes of CGN, which are rarely reported.

Therefore, we proposed a scientific hypothesis that κ-CGN in different solvents (aqueous and casein) may cause different degrees of intestinal mucus barrier damage due to differences in sulfate groups. In this study, κ-CGN was used as the research object and we aimed to investigate the regulatory effects of κ-CGN with different sulfate group exposures on gut microbiota and intestinal barrier with aqueous solution and casein in milk as the matrix, respectively. This result of our study provides a theoretical basis upon which to determine the safety of κ-CGN consumption.

## 2. Materials and Methods

### 2.1. Reagents

κ-CGN (Mw: 490 kDa, purity of 98%, sulfuric content of 22.15%) was purchased from Solarbio Technology (Beijing, China). Casein (casein sodium salt from bovine milk) was analytically pure and obtained from Sigma-Aldrich (St. Louis, MO, USA). The hematoxylin-eosin (HE) stain and alcian blue-periodic acid-Schiff (AB-PAS) dyeing solution set was purchased from Nanjing jiancheng Technology Co., Ltd. (Nanjing, China). The serum QuantiCyto Mouse interleukin (IL)-10, tumor necrosis factor (TNF)-α, and C-reaction protein (CRP) enzyme-linked immunosorbent assay (ELISA) kit were purchased from NeoBioscience Technology Co., Ltd. (Shenzhen, China). The Diaminobenzidine (DAB) kit was purchased from Fuzhou Maixin Technology Co., Ltd. (Fuzhou, China). Rabbit antibodies against zonula occluden (ZO)-1 were brought Proteintech Group, Inc. (Wuhan, China). Horseradish peroxidase (HRP)-conjugated goat antirabbit Immunoglobulin G (IgG) and goat antirabbit glyceraldehyde-3-phosphate dehydrogenase (GAPDH) were supplied by Nanjing KeyGen Biotech. Co., LTD. (Nanjing, China). 

### 2.2. Preparation of κ-CGN and κ-CC

κ-CGN solution (sulfuric acid content ≤ 20.52% [27], 1 g/L, 5 g/L, and 10 g/L) was prepared by dissolving κ-CGN powder in deionized water and stirred (800 rpm) for 30 min at 60 °C. Different concentrations of κ-CC complexes (sulfuric acid content ≤ 6.77% [27], 1 g/L, 5 g/L, and 10 g/L) were prepared by dissolving different weights of κ-CGN powder in 3% casein stock solutions and stirred (800 rpm) for 30 min at 60 °C. Dose levels selected for the study were 1 g/L, 5 g/L, and 10 g/L CGN. The low dose (1 g/L) is the concentration of CGN added in commercial hydrolyzed protein products and amino acid special formulas. The middle dose (5 g/L) is the concentration of CGN added in most milks and the high dose (10 g/L) is often used to induce intestinal barrier damage in animal models [5,11,28,30]. Casein is the key protein in bovine milk, accounting for about 3% [31].

### 2.3. Animals and Experimental Design

Male, six-week-old Kunming mice (weight 25–30 g) were obtained from the Beijing Huafukang Biotechnology Co., Ltd (Beijing, China). [license No. SCXK (Beijing) 2019-0008]. Mice were housed individually in clear white and movable cages with distilled water and diets. Environmental conditions included a 12 h light/dark cycle, with an average temperature targeted around 25 °C. After acclimatization for 7 days, mice were assigned to seven treatment groups and one control group with eight animals each based on a randomization program. The control group (CK1) was gavaged with normal ultrapure water. The treatment groups were given different concentrations of κ-CGN, casein, and κ-CC (1 g/L κ-CGN, CGNL; 5 g/L κ-CGN, CGNM; 10 g/L κ-CGN, CGNH; 3% casein, CK2; 1 g/L κ-CC, KCCL; and 5 g/L κ-CC, KCCM or 10 g/L κ-CC, KCCH). The average measurement method was adopted, and the gavage measurement was calculated according to 0.01 µL·kg^−1^·d^−1^. During the feeding period, the gavage dose was adjusted based on changes in mouse body weight: for every 0–10 g increase in weight, the gavage dose was increased by 0.05 mL accordingly. The experiment lasted 8 weeks, when mice were euthanized by cervical dislocation. The mice were all male and the sex was not associated with experimental studies. All experimental procedures were agreed on by the animal ethics committee of Quanzhou normal University (approval number QZTC-QZSYLL202207) and carried out in accordance with the *National Research Council’s Guide for the Care and Use of Laboratory Animals* (NIH Publication, 8th edition, 2011).

### 2.4. Sample Collection

Prior to mouse sacrifice, blood samples were collected and placed at room temperature for 0.5 h and then centrifuged at 4000 r/min for 10 min to obtain serum, which was stored at −80 °C until analysis. Fresh colon and spleen samples were placed directly into 0.01 M sterile phosphate buffered saline (PBS), which was precooled in advance. After cleaning, samples were placed in cryopreservation tubes and stored at −80 °C for later use. The luminal contents (stool) were collected from the colon with clean and sterilized forceps. The feces were placed into 1.5 mL centrifuge tubes and immediately placed at −80 °C for preservation.

### 2.5. Disease Activity Index (DAI) Assessment

The weight and feed of rats were recorded weekly. The DAI score was calculated to reflect the severity of barrier damage in mice, with the DAI criteria slightly adjusted to the actual situation of the experiment [32]. The scoring system included body weight, hair condition, activity, and stool character (Table 1).

### 2.6. Organ Index and Colon Length

Excess water was removed with absorbent paper and the weight of immune organs recorded. The organ coefficient was calculated as follows:

$$\mathrm{Organ}\;\mathrm{index}\;(\%)\;=\frac{\mathrm{Tissue}\;\mathrm{weight}}{\mathrm{Body}\;\mathrm{weight}}\times \;100\%$$

Colon length, an indicator of intestinal barrier damage, was measured from the proximal rectum to the ileocecal junction.

### 2.7. Serum Analysis

Based on the manufacturer’s instructions, the cytokine levels of serum IL-10, TNF-α, and CRP were analyzed using commercial enzyme-linked immunosorbent assay (ELISA) kits with a microplate reader (Multiskan FC, ThermoFisher Scientific, Waltham, MA, USA).

### 2.8. Histological Analysis

The colon was fixed in 4% paraformaldehyde, embedded in paraffin, and washed in absolute ethanol, 75% ethanol, and xylene in sequence. The procedures of Hematoxylin-Eosin (HE) and Alcian blue-periodic acid Schiff (AB-PAS) staining were operated according to the manufacturer’s instructions. The severity of intestinal barrier damage was assessed by calculating the histological activity index (HAI) under a 400x microscope (Olympus BX63, Tokyo, Japan) (Table 2). The final score was the sum of the barrier disruption area scores obtained from all pictures taken to represent the overall situation. In addition, the ratio of the area of the blue area compared to the full area of colon tissue was used to semi-quantitatively analyze the secretion of acidic mucus.

### 2.9. Quantitative Real-Time PCR (qRT-PCR)

Total RNA, which was extracted from the frozen colon tissues using TRIzol reagent (Code No. 15596-026; Invitrogen, Carlsbad, California, USA), was reverse transcribed into cDNA using Reverse Transcriptase RNase (Code No. RR036B; TaKaRa Bio, Tokyo, Japan). qPCR was performed on an ABI Veriti 96-well Thermal cycler PCR (Step one plus real-time-PCR system, Applied Biosystems, Waltham, MA, USA) using a qPCR SYBR Green Master Mix (Code No. 11201ES08; Yeasen, Shanghai, China). Each sample was assessed in triplicate and normalized to GAPDH. The primer sequences used are listed in Table 3.

### 2.10. Immunohistochemistry Staining

For examining the expression of mucus protein MUC2, the paraffin-embedded colon specimen was deparaffinized and rehydrated with dimethyl-benzene and alcohol. The sections were blocked by 3% H_2_O_2_. After incubated with the primary antibody anti-MUC2 rabbit (37 °C, overnight), we added HRP-conjugated goat anti-rabbit IgG as the secondary antibody. Finally, DAB developer was added to the solution. All sections were scanned with an optical microscope (Olympus BX63, Tokyo, Japan).

### 2.11. Western Blot Analysis

Briefly, the colon tissues were lysed with radio immunoprecipitation assay (RIPA) lysis buffer containing 0.1% protease, 1% phosphatase inhibitors, and 1% 100 mMPMSF and 12,000 g at 4 °C for 5 min to collect lysate supernatants. The total protein concentrations in the supernatants were quantified using the bicinchoninic acid (BCA) assay. Then, a polyacrylamide gel (6%) of the appropriate concentration for electrophoresis was prepared and transferred onto the polyvinylidene fluoride (PVDF) membrane. The membranes were blocked with 5% skimmed milk and incubated with primary rabbit monoclonal antibodies against ZO-1 (1:5000; Code No.21773-1-AP, Proteintech Group, Inc., Wuhan, China) and glyceraldehyde-3-phosphate dehydrogenase (CAPDH, 1:5000 dilution; Code No.KGAA002, KeyGen Biotech. Co., LTD, Nanjing, China) overnight at 4 °C. After being washed with 1× tris-borate-sodium tween (TBST) (10 mMTris, 150 mM NaCl, 0.1% Tween-20), the PVDF membranes were incubated with the horseradish peroxidase (HRP)-linked secondary antibodies (Code No.KGAA035, KeyGen Biotech. Co., LTD, Nanjing, China) for 1 h. Finally, the images were observed on the screen of the ChemiDoc MP Imaging System (Bio-Rad Gel Imaging Systems, Hercules, CA, USA), grayscale analysis was performed using Gel-Prosoftware (analyzer 4.0), and the experimental results were quantified according to the internal reference protein.

### 2.12. Fecal Microbial Community Analysis

Mouse feces were weighed, and the microbial genome extracted with a Magnetic Soil and Stool DNA kit (TIANGEN, Beijing, China). The Qubit dsDNA Assay Kit and a Qubit 2.0 Fluorimeter (Life Technologies, Carlsbad, CA, USA) were used to measure the concentration of obtained DNA. The quality of metagenomic DNA was assessed in 1% agarose gels. The DNA sample was fragmented by sonication to a size of 350 bp, then DNA fragments were end-polished, A-tailed, and ligated with full-length adaptors for Illumina sequencing and PCR amplification. After PCR amplification, the products were purified (AMPure XO system, Beckman coulter, Bray, CA, USA), libraries analyzed for size distribution with an Agilent 2100 Bioanalyzer, and quantified using real-time PCR (Bio-Rad CFX96, Hercules, CA, USA).

### 2.13. Statistical Analysis

All data were presented as means ± standard error (SEM) for each group. Statistical analyses were performed using SPSS 22.0 (Chicago, IL, USA) and visualized using GraphPad Prism 8.0 (San Diego, CA, USA) or Origin 2022. Independent samples *t*-tests were used for a single comparison of differences between groups and multiple comparisons were performed using one-way analysis of variance (ANOVA) with Turkey tests to determine statistical significance within the groups. Comparisons of relative abundances of microbiota at each level were carried out by Kruskal–Wallis H tests among three groups and Wilcoxon rank-sum tests between two groups [33]. Spearman’s rank correlation coefficient was used to assess the relationships among the gut microbiota and biochemical parameters. Uppercase letters represented differences within the κ-CGN groups, lowercase letters represented differences within the κ-CC groups, and “*” represented differences between the κ-CGN and κ-CC groups. Differences with *p* < 0.05 and *p* < 0.01 were considered statistically significant.

## 3. Results

### 3.1. Effects of κ-CGN and κ-CC on Clinical Symptom in Mice

**Figure 2:**
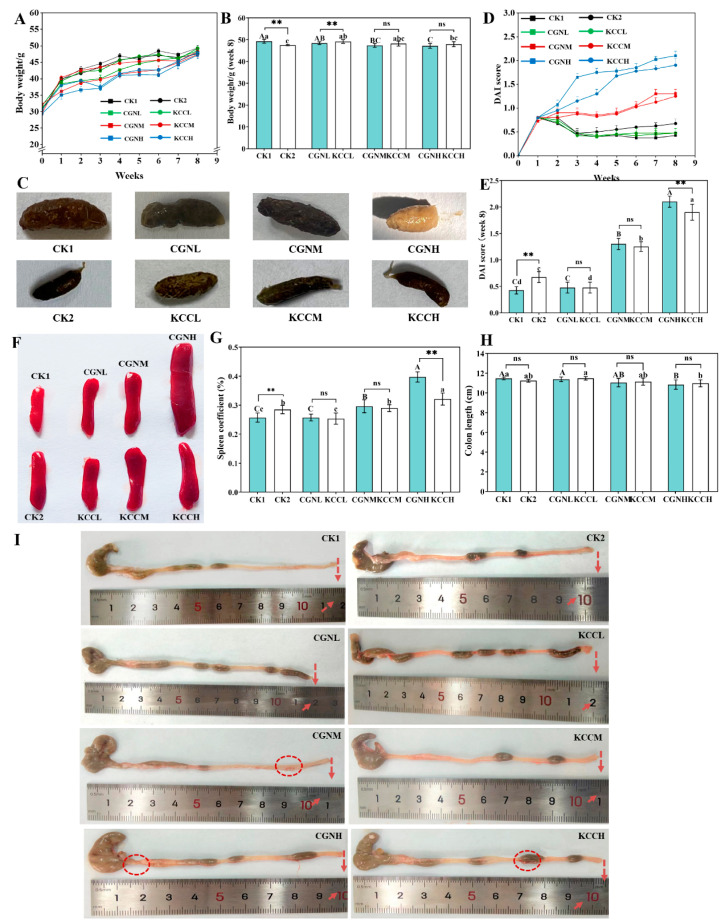
Effect of κ-CGN and κ-CC on colitis in mice (n = 8 for each group). (**A**) Body weight change from week 1 to 8. (**B**) Body weight change at week 8. (**C**) Fecal condition. (**D**) DAI scores change from week 1 to 8. (**E**) DAI scores change at week 8. (**F**) Spleen changes. (**G**) Spleen organ index at week 8. (**H**) Colon length change at week 8. (**I**) Colon condition. The arrows indicate the length of the colon, and the circles indicate the occurrence of congestion. Independent samples *t*-tests were used for a single comparison of differences between groups and multiple comparisons were performed using the Turkey post hoc test after a significant one-way ANOVA (*p* < 0.05). Uppercase letters represent differences within the κ-CGN groups, lowercase letters represent differences within the κ-CC groups. “*” represents differences between the κ-CGN and κ-CC groups (*p* < 0.05) and “**” represents differences between the κ-CGN and κ-CC groups (*p* < 0.01).

To explore the effect of κ-CGN in different matrices (aqueous and 3% casein) on clinical symptoms in mice, the change in body weight, DAI scores, spleen organ index, and colon length were examined. As shown in Figure 2, compared with the CK1 group, there was no significant difference in body weight, DAI scores, spleen organ index, and colon length in κ-CGN and κ-CC mice at a low dose (*p* > 0.05). However, with the increase in dose, the pathological indicators showed obvious lesions (*p* < 0.05). In addition, the mice in the CGNH group exhibited loose, yellowish stools, and CK2 as well as KCCH groups showed mucosal attachment on the outside of the stool. Compared to κ-CGN groups, the κ-CC groups attenuated κ-CGN-induced body weight loss, DAI scores, and had smaller organ coefficients (*p* < 0.05). These data reveal that more than 5 g/L of κ-CGN in aqueous solution and in 3% casein had side effects, but the symptoms were milder for the κ-CC groups where casein was the vehicle.

During feeding of the medium-dose groups, fighting interfered with the analysis of results, so results for the medium-dose groups were rounded out in subsequent analysis.

### 3.2. Effects of κ-CGN and κ-CC on Inflammatory Cytokines in Mice

**Figure 3:**
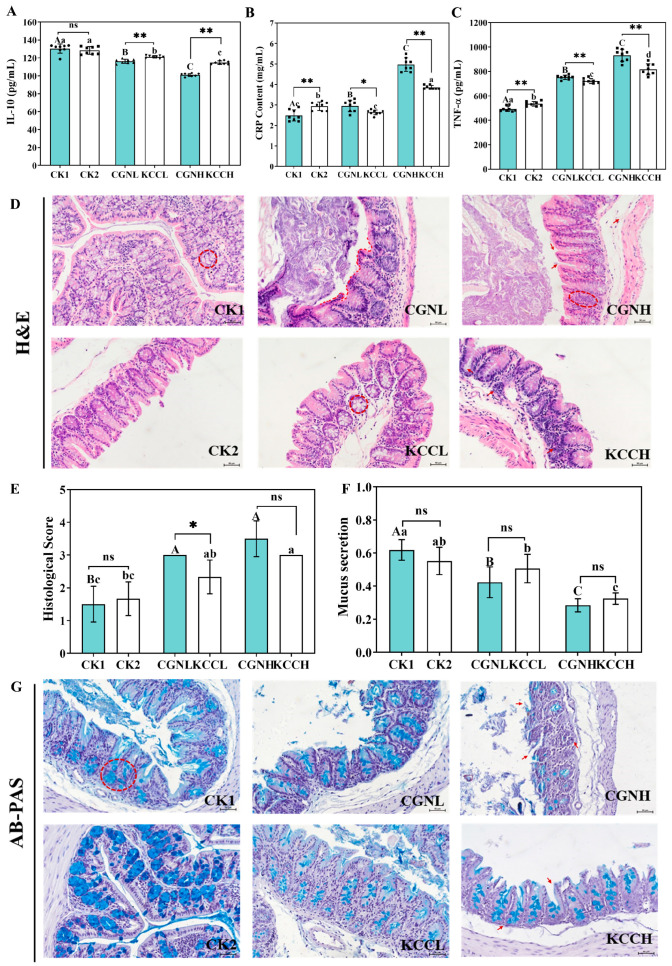
Effects of κ-CGN and κ-CC on inflammatory cytokines and the intestinal barrier (n = 8 for each group). (**A**–**C**) Serum inflammatory cytokines levels of TNF-α, CRP, and IL-10. (**D**) Images of HE staining. The dotted line indicates the surface of the irregular crypt and arrows indicate infiltration of inflammatory cells. (**E**) HAI scores. (**F**) Quantification of mucus secretion. (**G**) Images of AB-PAS staining. Circles and arrows indicate acidic mucus staining. Independent samples *t*-tests were used for a single comparison of differences between groups and multiple comparisons were performed using the Turkey post hoc test after a significant one-way ANOVA (*p* < 0.05). Uppercase letters represent differences within the κ-CGN groups, lowercase letters represent differences within the κ-CC groups. “*” represents differences between the κ-CGN and κ-CC groups (*p* < 0.05) and “**” represents differences between the κ-CGN and κ-CC groups (*p* < 0.01). scale bar = 50 μm.

The concentrations of pro-inflammatory cytokines CRP, TNF-α, and anti-inflammatory cytokine IL-10 in the serum were assessed (Figure 3A–C). Firstly, the CK2 group was slightly decreased compared to the CK1 group, with no significant difference observed (*p* > 0.05). A remarkable reduction, in a dose-dependent manner, for IL-10 was observed in κ-CGN and κ-CC groups (*p* < 0.05). As expected, both KCCL and KCCH induced higher levels of anti-inflammatory cytokine secretion than did κ-CGN treatment (*p* < 0.05). In addition, despite κ-CGN and κ-CC groups both inducing a significant increase in CRP and TNF-α compared to CK1, it is worth noting that both KCCL and KCCH reversed the increase in pro-inflammatory cytokines induced by κ-CGN under the equivalent concentrations (*p* < 0.05).

### 3.3. Effect of κ-CGN and κ-CC on Intestinal Barrier in Mice

HE staining and AB/PAS staining were used to observe damage and acid mucin secretion in the colonic tissue sections. As shown in Figure 3D–G, with increasing doses, the treatment of κ-CGN and κ-CC showed irregular crypt surface, higher HAI scores, and lower secretion of mucin (*p* < 0.05). The epithelial structure of the CK2 group was intact and there was no significant difference from that of the CK1 group. Moreover, compared to CGNL groups, the KCCL group had significantly decreased HAI scores.

**Figure 4:**
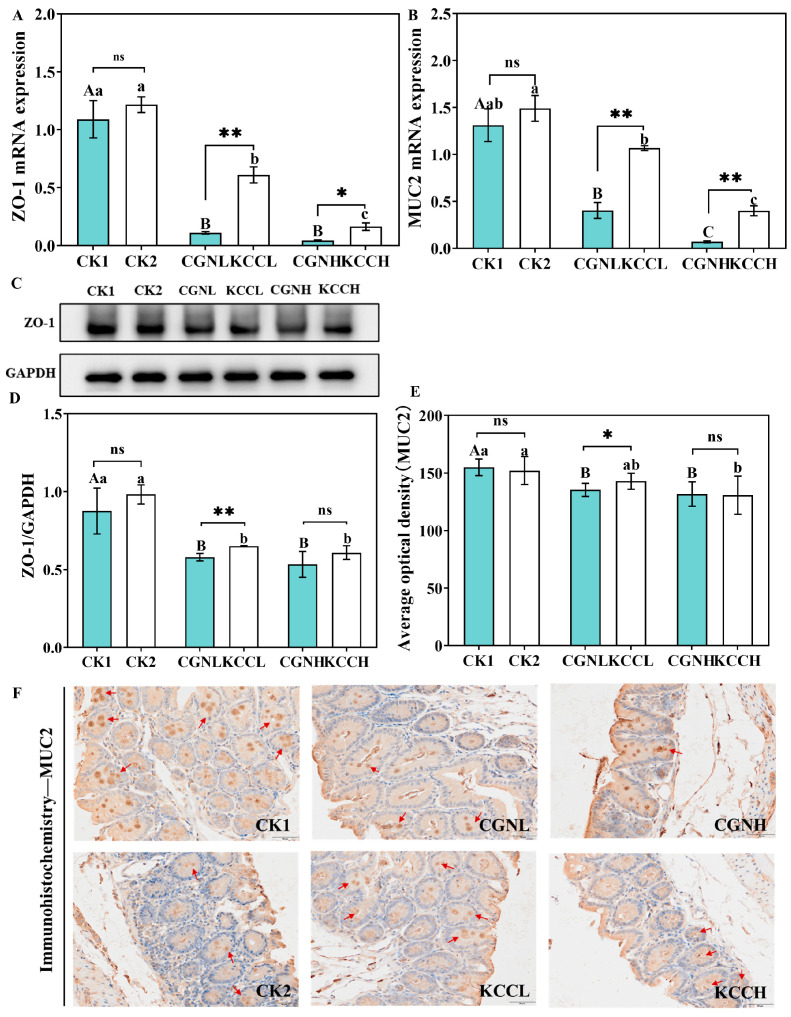
Effect of κ-CC and κ-CGN on the expression of ZO-1 and MUC2 in mice (n = 8 for each group). (**A**,**B**) The mRNA levels of ZO-1 and MUC2. (**C**,**D**) Western bolt results and the protein expression of ZO-1. (**E**) The protein expression of MUC2. (**F**) Immunohistochemistry staining. The arrows indicated MUC2 staining. Independent samples *t*-tests were used for a single comparison of differences between groups and multiple comparisons were performed using the Turkey post hoc test after a significant one-way ANOVA (*p* < 0.05). Uppercase letters represent differences within the κ-CGN groups, lowercase letters represent differences within the κ-CC groups. “*” represents differences between the κ-CGN and κ-CC groups (*p* < 0.05) and “**” represents differences between the κ-CGN and κ-CC groups (*p* < 0.01). scale bar = 50 μm.

Defects in intestinal barrier function cause intestinal inflammatory disease, so we examined the tight junction protein and mucus protein expression [34]. As shown in Figure 4A,B, the treatment of κ-CGN in aqueous as well as casein reduced the mRNA levels of ZO-1 and MUC2 in a dose-dependent manner (*p* < 0.05). Notably, the expression of junction or mucus protein in κ-CC groups significantly increased compared to κ-CGN groups (*p* < 0.05). Additionally, the Western blot and immunohistochemistry staining results (Figure 4C–F) demonstrate that the expression levels of ZO-1 and MUC2 were significantly decreased in κ-CGN and κ-CC groups, and κ-CC-treatment increased the expression of ZO-1 and MUC2 compared to κ-CGN groups (*p* < 0.05). Equally with the HAI scores, there was a more significant relief effect under the condition of the low-dose group in terms of barrier damage.

### 3.4. Effect of κ-CGN and κ-CC on the Structure and Diversity of Gut Microbiota in Mice

**Figure 5:**
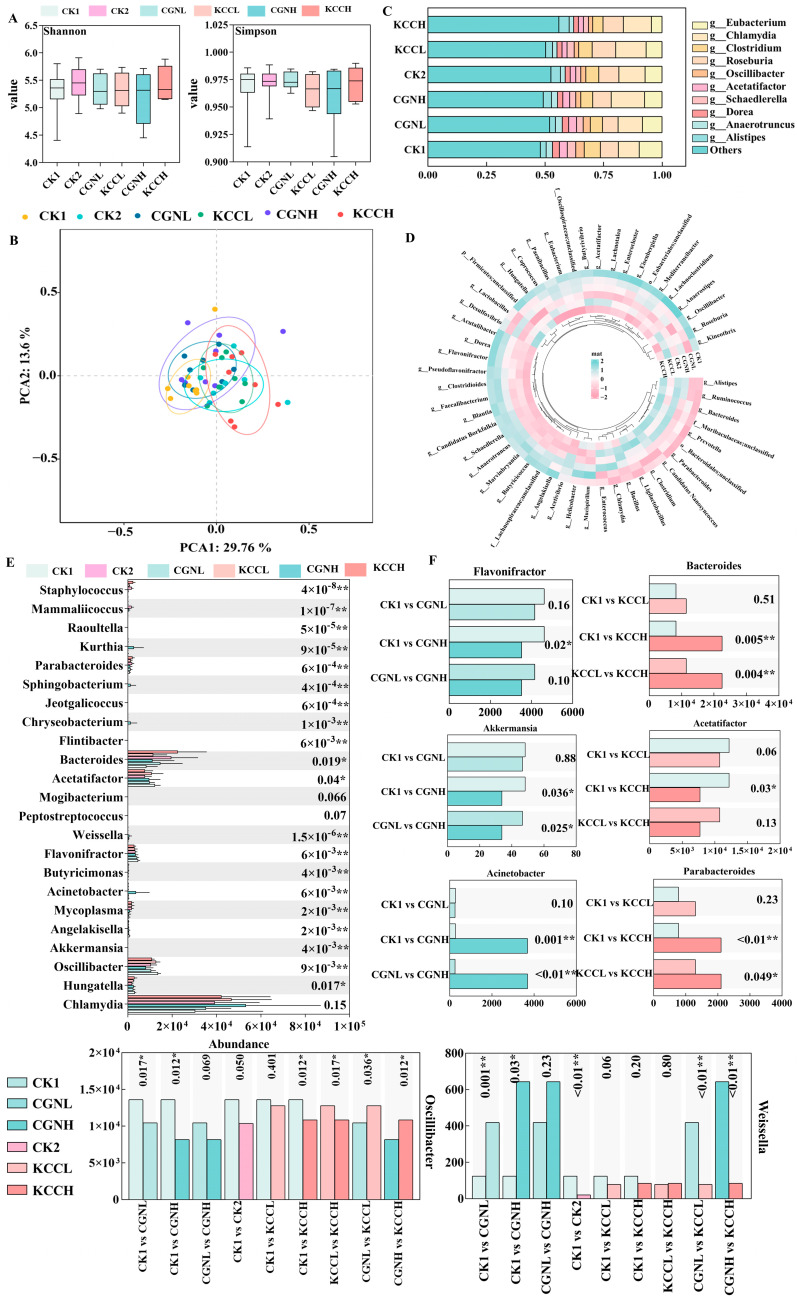
Effect of κ-CGN and κ-CC on the gut microbiota at genus (n = 8 for each group). (**A**) Alpha diversity. (**B**) Beta diversity. (**C**) Stacked column plot of microbial genus relative abundance. (**D**) Heatmap analysis of relative abundance of top 50 genera. (**E**) The Kruskal–Wallis test results for comparison of microbial abundance among six groups. (**F**) Relative abundance of differential bacteria in κ-CGN and κ-CC groups, which were calculated by a Wilcoxon rank sum test, * *p* < 0.05, and ** *p* < 0.01.

As shown in Figure 5A, Shannon and Simpson indices showed there is no significant differences in microbiota diversity and richness was observed in the κ-CGN and κ-CC groups. A PCA diagram (Figure 5B) shows that the CK1 group was distinct from CGNH group and KCCH group. No significant separation of microbial composition was found for the CGNH and KCCH groups or the CGNL and KCCL groups. In addition, individual samples of the CK1 group clustered together with good reproducibility. However, with high-dose κ-CGN, individual samples were scattered with large differences, indicating that CGN reduced the stability of the intestinal flora of mice.

### 3.5. Effect of κ-CGN and κ-CC on the Composition and Abundance of the Gut Microbiota

*Chlamydia* and *Oscillibacter* were the major genera with κ-CGN and κ-CC, but the proportions were different (Figure 5C). Compared with other groups, the proportion of *Oscillibacter* in the control group was relatively high. To visualize the changes in microbiota diversity after treatment of κ-CGN and κ-CC, the relative abundance of 50 genera were compared using a heat map (Figure 5D). Hierarchical differences in the heat map analysis showed that the κ-CGN relative abundance patterns were clearly distinct from that of κ-CC.

The Kruskal–Wallis significant difference test was used to explore differences within six groups (Figure 5E) and the Wilcoxon rank-sum test was further used to explore differences between the two groups (Figure 5F). In regard to κ-CGN groups, the harmful bacteria *Actinetobacter* was enriched in CGNH groups. Both CGNL and CGNH had a significantly greater abundance of pathogenic bacteria *Weissella* as well as *Chryseobacterium*. In addition, the abundance of beneficial microflora *Oscillibacter*, *Flavonifractor*, *Akkermansia*, and *Flintibacter* in CGNH was lower than CK1 group (*p* < 0.05). Within κ-CC groups, probiotics of *Oscillibacter* and *Acetatifactor* decreased gradually and showed difference in KCCH group. Both KCCL and KCCH exhibited significant decrease in beneficial microflora *Blautia,* with an increase in the proportion of pathogenic *Bacteroides*, *Parabacteroides,* and *Brucella* (*p* < 0.05). However, compared to the CGNL group, we found decreased levels of *Weissella* and *Jeotgalibacillus*, but increased levels of *Oscillibacter* and *Mammaliicoccus* in KCCL-fed mice. Compared to the CGNH group, the relative abundance of *Weissella* and *Jeotgalicoccus* decreased, while *Oscillibacter* and *Rhodobacter* increased in the KCCH group.

**Figure 6:**
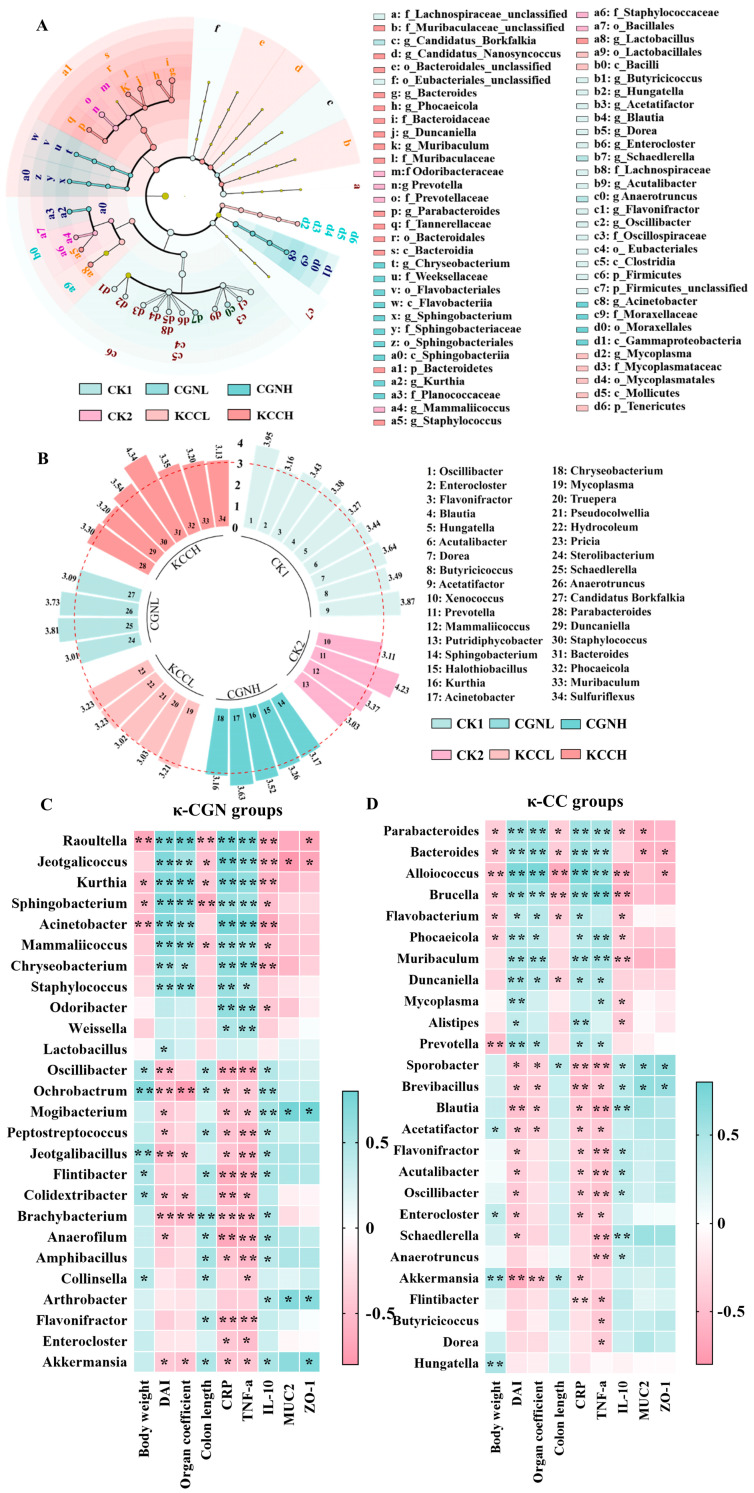
LEfSe analysis of gut microbiota and Spearman’s analysis between the microbiota and biochemical indexes. (**A**) Taxonomic cladogram obtained from LEfSe analysis among six groups. Different colors indicate the enrichment of the biomarker taxa. The circle from inside to outside means the rank from kingdom to genus, and the circle size represents the taxa abundance in the community. (**B**) Circle bar of LDA scores from LEfSe analysis at genus (LDA > 3). (**C**) Correlation analysis of characteristic microbiota and biochemical indexes in the κ-CGN group. (**D**) Correlation analysis of characteristic microbiota and biochemical indexes in the κ-CC group. The color scale represents the strength of correlation, ranging from 0.5 (strong positive correlation) to − 0.5 (strong negative correlation). * *p* < 0.05, ** *p* < 0.01.

Changes in the gut microbiome and prognostic biomarkers of microbial abundance for the six different groups of mice were assessed by linear discriminant analysis effect size (LEfSe) analysis (https://huttenhower.sph.harvard.edu/lefse/ accessed on 17 December 2024), which was performed for taxa by linear discriminant analysis (LDA) (LDA > 3) (Figure 6A,B). As shown in Figure 6B, probiotics such as *Oscillibacter*, *Acetatifacter*, *Dorea*, *Acutalibacter,* and *Flavonifractor* were more abundant in the CK1 group. Treatment with CGNL increased the abundance of *Anaerotruncus* and *Schaedkerella,* and the pathogenic microbiota *Acinetobacter* and *Kurthia* were enriched in the CGNH group. *Hydrocoleum* was more abundant in the KCCL group and the levels of *Bacteroides*, *Parabacteria,* and *Phocaeicola* were enriched in the KCCH group.

### 3.6. The Correlation Among Biological Parameters and the Gut Microbiota

In order to explore whether these abundance changes of differential bacteria in the κ-CGN and κ-CC groups were associated with the clinical symptoms and intestinal barrier damage, we performed Spearman’s correlation analysis between the abundance of differential bacteria and pathological symptoms (Figure 6C). For κ-CGN in aqueous solution groups, we found an increased abundance of the pathogenic bacterium *Acinetobacter*, which was positively associated with higher DAI scores, organ coefficient, and the secretion of pro-inflammatory cytokines, but negatively associated with body weight and levels of IL-10. Probiotics *Oscillibacter* and *Flavonifractor*, which had a lower abundance in κ-CGN groups, were negatively proportional to TNF-α and to CRP, but positively proportional to colon length. *Akkermansia* had greater anti-inflammatory characteristics and had closely positive correlation with the secretion of IL-10 and colon length, but negative correlation with spleen coefficient. Additionally, several opportunistic pathogens, *Raoultella* and *Jeotgalicoccus,* have a closely negative correlation with tight junction protein (ZO-1) and mucin protein (MUC2) in the intestinal barrier. For κ-CGN in casein groups, the higher levels of *Bacteroides* and *Parabacteroides* were related to higher DAI scores, spleen coefficient, and the level of pro-inflammatory cytokines, but had negative correlation with the ZO-1 and MUC2. The probiotics *Oscillibacter* and *Acetatifactor* were inversely proportional to DAI scores as well as pro-inflammatory cytokines. In addition, beneficial microflora *Blautia*, which had a lower abundance in κ-CC groups, was negatively proportional to DAI score, spleen coefficient, CRP, and TNF-α, but positively associated with the secretion of IL-10.

## 4. Discussion

CGN is a highly sulfated polysaccharide, which is generally divided into κ-, β- and ɩ-CGN types according to the amount and position of sulfate groups. κ-CGN contains approximately 25–30% of ester sulfate content [22]. More and more studies indicated that sulfate is closely related to intestinal barrier damage as well as microbiota [35]. For example, Prof. Luo pointed out in 2024 that gut microbiota can degrade sulfopolysaccharides and assimilate sulfates to produce hydrogen sulfide through the assimilatory sulfate reduction pathway. People are reminded not to use sulfopolysaccharides as food additives [26]. Our previous studies showed that the free sulfate group’s content was always lower in κ-CGN dissolved in casein (≤6.77%) than κ-CGN in aqueous solution (≤20.52%) [27]. Additionally, the difference in the content of sulfate groups of κ-CGN may have an impact on the physiological function and intestinal barrier function of mice. Therefore, this study dissolved κ-CGN in different solvents (aqueous and casein) to explore the relationship between sulfate exposure and intestinal barrier homeostasis.

Body weight, DAI scores, spleen coefficient, and colon length are classic indictors of intestinal barrier damage [36]. In our study, low-dose κ-CGN and κ-CC showed no significant change compared to the control group in body weight, DAI scores, spleen coefficient, and colon length. The phenomenon indicated that 1 g/L κ-CGN in different solvents (aqueous or casein) did not exhibit inflammatory effects, which was consistent with most previous studies, especially the experimental results of Weiner et al. [11]. With the increase in dose, weight loss, higher DAI score, higher spleen coefficient, and shorter colon length were induced by κ-CGN and κ-CC. Interestingly, the pathological symptoms were milder in κ-CC groups where casein is the vehicle under the equivalent concentrations. We can deduce that less exposure of sulfate groups may have a regulation effect on the degree of pathological dysfunction in mice.

To further assess the degree of intestinal barrier damage in mice, we measured inflammatory cytokines in serum. TNF-α, CRP, and IL-10 are closely related to the mucosal immune system and UC [37,38,39]. Elevated TNF-α and CRP levels could stimulate the production of other cytokines, arachidonic acid metabolites, and proteases, which amplify the inflammatory response [40,41,42,43,44]. Our results show that both κ-CGN dissolved in both aqueous solution and casein led to a significant increase in the levels of TNF-α and CRP, and a decrease in the levels of IL-10 in a dose-dependent manner. IL-10 is principally produced by Treg cells, which dampen cell-mediated and humoral immune responses, inhibiting the production of a variety of pro-inflammatory cytokines, such as TNF-α and IL-6 [45,46]. It should be noted that we found κ-CGN dissolved in casein significantly decreased the expression level of TNF-α and CRP, and increased the levels of IL-10 compared to an equivalent dose of κ-CGN aqueous solution, as well as inhibited the development of intestinal barrier damage. This indicated that κ-CGN in casein with a less free sulfate group effectively reduced the proliferation of pro-inflammatory factors and inhibited the expansion of the inflammatory area. The increase in inflammatory factor levels in low-dose κ-CC is not consistent with the experimental results of Weiner et al. [11], which may be due to the difference in maturity of pre-weaning piglets and adult mice as well as differences in feeding cycles (4 weeks in Weiner, but 8 weeks in our research).

Epithelial barrier integrity plays an important role in maintaining intestinal immune homeostasis [47]. ZO-1 is the most important component of tight junctions, as they can prevent the invasion of harmful substances through tight connections [48]. Multiple instances of evidence suggest that defects in the mucus layer or the absence of MUC2 protein may contribute to intestinal barrier damage [49,50]. In agreement with previous studies [8,30,49], we confirmed that the mRNA and protein expressions of ZO-1 and MUC2 in κ-CGN and κ-CC were lower than the control group in a dose-dependent manner. It was worth noting that our results suggest κ-CGN in casein groups attenuated the loss of mucus secretion and the destruction of an intestinal barrier induced by κ-CGN aqueous solution. Previous studies proposed that the change in the viscosity of soluble fiber during gastrointestinal digestion has an impact on its biological effect. Lower contents of sulfuric acid group exposure can reduce the viscosity of dietary fiber and polysaccharides [51,52,53]. The reduction in viscosity may reduce the contact with the intestinal mucosa, thereby reducing the damaging effect on the intestinal barrier [54,55]. Therefore, we believed that κ-CGN in 3% casein with less free sulfate group can reduce the contact between κ-CGN and the intestinal epithelium by reducing the exposure of sulfate groups, thereby increasing the secretion of mucus and alleviating the disrupting effects of κ-CGN on the intestinal barrier status in mice.

**Figure 7:**
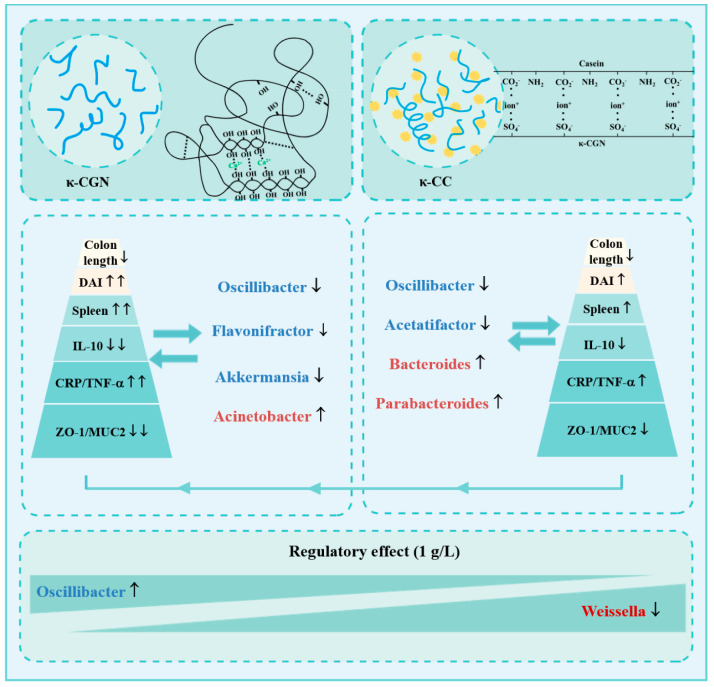
Schematic diagram of κ-CGN solution and κ-CC causing microbiota changes in mice. (The arrows indicate upward and downward changes in microbiota or physiological indicators.)

The gut microbiota, as a biological barrier, is an important part of intestinal barrier in mice [56]. In this study, we found four characteristic bacteria associated with intestinal barrier homeostasis induced by κ-CGN aqueous solution. One of them is a pathogenic bacterium, *Acinetobacter*, and three probiotics, *Oscillibacter*, *Flavonifractor*, and *Akkermansia* (Figure 7); firstly, the harmful bacteria *Acinetobacter*, which significantly increased in the CGNH group and was positively related to disruption of mucus barrier. Many researchers reported *Acinetobacter* was enriched in DSS-induced epithelial barrier disruption and closely associated with the progression of barrier damage [57,58]. Then, the abundance of *Oscillibacter* and *Flavonifractor* was significantly decreased in CGNH groups and negatively related to barrier damage. *Oscillibacter* has been reported to downregulate the activation of NF-κB and upregulate the differentiation of IL-10-producing Treg cells through producing SCFAs [59,60]. Furthermore, *Flavonifractor plautii* also plays a protective role in disruption of the gut barrier, which can be involved in the metabolism of catechins in the human gut and alleviate mucosal injury by suppressing the overexpression of the inflammatory factor IL-17 [61]. Finally, it was interesting that our results also show a decreasing trend in *Akkermansia* in the high-dose group (10 g/L), which was consistent with the previous research [18]. *Akkermansia*, a potent anti-inflammatory intestinal bacterium, can adhere to intestinal cells and enhance the integrity of the epithelial cell layer [62,63,64].

Equally with κ-CGN in aqueous solution, we identified four characteristic bacteria associated with intestinal barrier homeostasis in κ-CC. Two of them are the pathogenic bacteria *Bacteroides* and *Parabacteroides*, and the other two are the probiotics *Acetatifactor* and *Oscillibacter. Bacteroides* and *Parabacteroides* are usually considered as pathogenic bacterium and are able to induce intestinal barrier damage in murine models [65,66]. *Bacteroides acidifaciens* and *Bacteroides fragilis* in members of *Bacteroides* are considered as mucin-degrading bacterium and destroy the intestinal epithelial layer barrier [67]. Meanwhile, the relative abundance of *Parabacteroides*, a new genus discovered in recent years, in the exacerbation period of UC increased significantly compared to the remission stage [68]. Both *Bacteroides* and *Parabacteroides* showed higher abundance in κ-CC groups and closely were associated with severe intestinal barrier damage in this study. Furthermore, the treatment of κ-CC reduced the relative abundance of probiotic, including *Acetatifactor* and *Oscillibacter*, resulting in an increase in pro-inflammatory factors. *Acetatifactor* and *Oscillibacter* are both producers of SCFAs, especially butyrate, with the ability to alleviate intestinal barrier damage induced by DSS [69,70].

A major finding is that a significantly higher abundance of the probiotic *Oscillibacter*, which negatively related to intestinal barrier damage, was observed in κ-CC-treated groups compared to κ-CGN groups under the same concentrations. In the previous research, *Oscillibacter* was known as a producer of anti-inflammatory metabolites, such as butyrate, and usually associated with a beneficial effect on gut barrier homeostasis [71]. In addition, the abundance of the harmful bacteria *Weissella* in any dose was significantly lower in κ-CC groups compared to κ-CGN mice. *Weissella*, as a new genus of lactic acid bacteria, has been shown to alleviate epithelial barrier damage by reducing gut permeability and attenuating disruption of the intestinal epithelial barrier in murine models [72,73,74,75]. However, in the early years, the Germany Committee for Biological Agents already appointed *Weissella confusa* as a classified Risk Group 1 microorganism [76]. In fact, *Weissella*, as a common opportunistic pathogen, was usually found to be predominant in the model of intestinal barrier damage in mice [77,78]. The virulence factors and lipoteichoic acids produced by *Weissella* have been shown to increase the secretion of pro-inflammatory cytokines, leading to host infections and diseases [79,80,81]. In line with the previous research, *Weissella* exhibited a closely positive correlation with the pro-inflammatory factors in this paper.

A diet rich in sulfates can influence the metabolism of the colon and is reduced by bacteria to toxic and harmful intestinal H_2_S [82]. The production and amount of H_2_S are cofactors of inflammatory bowel disease. An increase in the concentrations of H_2_S can disrupt the balance between cellular proliferation and apoptosis, block SCFAs metabolism, and lead to the development of inflammatory damage to the intestinal epithelium [82,83]. In our study, κ-CC groups with a lower sulfate group content increased in relative abundance of the butyrate-producing bacteria *Oscillibacter*, which might increase the secretion of SCFAs compared to κ-CGN groups. Of course, research on fecal metabolomics needs to be further performed to clarify this speculation. From this, we can know that κ-CGN in casein might help relieve the occurrence of intestinal barrier damage induced by long-term (8 weeks) treatments of κ-CGN aqueous solution through increasing the abundance of *Oscillibacter* and decreasing the abundance of the pathogenic bacterium *Weissella*.

## 5. Conclusions

As a food additive, κ-CGN is added to various foods in the range of about 0.01–0.5% (0.1–5 g/L), such as dairy products, meat products, and jell (https://www.foodsweeteners.com, accessed on 14 October 2023). In this study, the results show 1 g/L κ-CGN in aqueous and casein does not have adverse effects on the growth and development of mice. As there is an increase in κ-CGN dose as well as the sulfate group exposure, the side effects (epithelial barrier damage) are obvious. In aqueous solution, high-dose long-term (8 weeks) treatments of κ-CGN (>1 g/L) have the potential to disrupt the intestinal barrier by increasing the abundance of pathogenic *Acinetobacter* and decreasing the abundance of *Oscillibacter*, *Flavonifractor*, and *Akkermansia*. In 3% casein solution, a high dose (10 g/L) of κ-CC provided a pro-inflammatory environment through increasing the abundance of harmful bacteria *Bacteroides* and Parabacteroides as well as decreasing the abundance of the probiotics Acetaifactor and *Oscillibacter.* However, more importantly, κ-CGN in casein with lower exposure of sulfate groups alleviated κ-CGN solution-induced colonic damage and mucus barrier degradation by increasing the abundance of *Oscillibacter*, decreasing the abundance of *Weissella*. The regulatory effects were reflected in the higher colon length, lower DAI scores and spleen coefficients, higher secretion of anti-inflammatory cytokines (IL-10), lower levels of pro-inflammatory cytokines (TNF-α), higher secretion of goblet cells, mucus, and raised expression of epithelial cell compact proteins ZO-1 as well as MUC2. In conclusion, the safety, intestinal barrier function, and microbiota homeostasis of κ-CGN dissolved in different food matrices (water and casein solutions) with different amounts of sulfate group exposure are different. In addition, these results remind us that the content of sulfate group exposure can be involved in normal intestinal barrier function homeostasis, which we will verify in future experiments.

## Figures and Tables

**Table 1:** Standard for evaluation of the disease activity index (DAI).

Score	0	1	2
Changes of body weight	1–5%	5–10%	10–15%
Hair condition	Neat gloss	Cluttered and shiny	Cluttered, sparse, dull
Activity	Normal	Inactive	Motionless
Stool color	Black	Yellow	White
Stool shape	Normal	Soft	Watery
Bloody stool	—	Few	obvious

**Table 2:** HAI scoring criteria.

Score	Epithelial Damage	Infiltration of Inflammatory Cells
0	Normal	None
1	A small loss of goblet cells	Infiltrate around the base of the crypt
2	Massive loss of goblet cells	Infiltration of the basal mucosa
3	A small loss of crypts and massive loss of goblet cells	extensively infiltrated and edematous of mucosa
4	Massive loss of crypts	Submucosal infiltration

**Table 3:** Primer sequences of qRT-PCR.

Target Gene	Forward Primer (5′→3′)	Revere Primer (5′→3′)
ZO-1	AAATCATCCGACTCCTCGTCG	GACAGAAACACAGTTGGCTCC
MUC2	GGCCAGGAGTTTACCAACGA	CAGGGCAAGGCAGGTCTTTA
GAPDH	CCCAGCTTAGGTTCATCAGG	CCAAATCCGTTCACACCGAC

## Data Availability

The original contributions presented in the study are included in the article, further inquiries can be directed to the corresponding author.

## Abbreviations

Carrageenan, CGN; κ-carrageenan, κ-CGN; κ-carrageenan-casein, κ-CC; Disease Activity Index, DAI; Tumor necrosis factor α, TNF-α; C-reaction protein, CRP; Interleukin 10, IL-10; Zonula occluden 1, ZO-1; Mucin protein 2, MUC2; Joint Expert Committee on Food Additives, JECFA; Hematoxylin-eosin, HE; Alcian blue-periodic acid-Schiff, AB-PAS; Enzyme-linked immunosorbent assay, ELISA; Horseradish peroxidase, HRP; Phosphate buffered saline, PBS; Histological activity index, HAI; Diaminobenzidine, DAB; Quantitative Real-Time PCR, qRT-PCR; Western Blot, WB; Polyvinylidene fluoride, PVDF; Bicinchoninic Acid, BCA; Radio Immunoprecipitation Assay, RIPA; Glyceraldehyde-3-phosphate dehydrogenase, GAPDH; Immunoglobulin G, IgG; Inflammatory bowel disease, IBD; Linear discriminant analysis Effect Size, LEfSe; Linear Discriminant Analysis, LDA; Lipopolysaccharide, LPS; Toll-like receptors 4, TLR4.
